# A Review of the Prevalence of Thromboembolic Complications among Pregnant Women Infected with COVID-19

**DOI:** 10.3390/jcm11195934

**Published:** 2022-10-08

**Authors:** Nurul Syafiqah Mohd Ariff, Izzati Abdul Halim Zaki, Zakiah Mohd Noordin, Nur Sabiha Md Hussin, Khang Wen Goh, Long Chiau Ming, Hanis Hanum Zulkifly

**Affiliations:** 1 Faculty of Pharmacy, Universiti Teknologi MARA (UiTM), Cawangan Selangor, Kampus Puncak Alam, Puncak Alam 42300, Malaysia; 2 Cardiology Therapeutics Research Group, Universiti Teknologi MARA, Puncak Alam 42300, Malaysia; 3 Faculty of Data Science and Information Technology, INTI International University, Nilai 71800, Malaysia; 4 PAP Rashidah Sa’adatul Bolkiah Institute of Health Sciences, Universiti Brunei Darussalam, Gadong BE1410, Brunei

**Keywords:** COVID-19, thromboembolic complications, venous thromboembolism, prevalence, disseminated intravascular coagulation, intervillous thrombosis

## Abstract

**Background:** Thrombotic conditions triggered by SARS-CoV-2 virus can result in high mortality, especially in pregnant women as they are already in a hypercoagulability state. This thereby leads to excessive inflammation that will increase the risk of thromboembolic (TE) complications. **Objective:** The aim of this study is to review the prevalence of thromboembolic complications such as deep venous thrombosis, pulmonary embolism, and intervillous thrombosis, and their preventive strategies among pregnant women infected with COVID-19. **Method:** The articles were retrieved from online databases PubMed and ScienceDirect published from February 2020 to April 2022. **Findings:** A total of 5249 participants including 5128 pregnant women and 121 placentas from 19 studies were identified for having TE complications after being infected with COVID-19. The types of TE complications that developed within pregnant women were disseminated intravascular coagulation (DIC) (*n* = 44, 0.86%), unmentioned thromboembolic complications (TE) (*n* = 14, 0.27%), intervillous thrombosis (IVT) (*n* = 9, 0.18%), pulmonary embolism (PE) (*n* = 6, 0.12%), COVID-19 associated coagulopathy (CAC) (*n* = 5, 0.10%), and deep venous thrombosis (DVT) (*n* = 2, 0.04%). Whereas the prevalence of TE complications reported from studies focusing on placenta were IVT (*n* = 27, 22.3%), subchorionic thrombus (SCT) (*n* = 9, 7.44%), and placental thrombosis (*n* = 5, 4.13%). Thromboprophylaxis agent used among pregnant women include low molecular weight heparin (LMWH) at prophylactic dose (*n* = 9). **Conclusions:** The prevalence of thromboembolic complications among pregnant women infected by COVID-19 is low with DIC being the most common form and placental thrombosis being the least common form of TE complications that occurred within pregnant women infected with COVID-19. Anticoagulation, in particular LMWH (variable dose), is frequently used to prevent TE complications.

## 1. Introduction

As of December 2019, Coronavirus Disease 2019 (COVID-19) is an infectious disease caused by a new coronavirus, SARS-CoV-2, which was first reported in Wuhan, China. It is a single-stranded RNA virus that infects cells by binding to angiotensin converting enzyme-2 (ACE2) receptors [1,2]. The infection may be asymptomatic or symptomatic with clinical manifestations such as fever, cough, sore throat, and headache, but it might also develop into severe diseases. Despite the fact that COVID-19 was initially thought to be a pulmonary sickness that caused morbidity and mortality in the elderly and those with underlying medical disorders, younger and pregnant patients may also develop moderate to severe symptoms that require hospitalization due to various complications such as thromboembolic (TE) complication.

TE complications are often multifactorial. Venous thromboembolism (VTE) is a disorder in which the blood clots abnormally and is linked to a high rate of morbidity and mortality [3]. VTE can be divided into deep vein thrombosis (DVT) and pulmonary embolism (PE). DVT occurs when clots are developed in the deep veins, primarily in the legs, while PE occurs when a clot in the deep vein lodges loosely and travels to the arteries of the lungs. VTE episodes were shown to be four- to five-folds higher in pregnant women than in non-pregnant women [4,5]. The clinical manifestations of DVT are usually swelling and pain in the extremity or lower leg regions, whereas PE is characterized by symptoms such as dyspnoea, tachypnoea, and chest pain.

Meanwhile, disseminated intravascular coagulation (DIC) is another TE complication, which is characterized by the presence of many micro clots in the vasculature, as well as an increased haemorrhagic predisposition [6]. The signs of DIC are usually bleeding, shortness of breath, and low blood pressure due to constant bleeding. COVID-19 associated coagulopathy (CAC) is also one of the TE complications that develop due to the fact that COVID-19 is highly prothrombotic in which it can cause overstimulation of inflammatory factors resulting in the coagulation cascade to occur.

Meanwhile, other types of TE complications can also develop in the placenta of pregnant women, e.g., (i) intervillous thrombosis (IVT), in which the blood clot is present in the intervillous space; (ii) subchorionic thrombus (SCT), in which the thrombus confined to the subchorionic space; and (iii) placental thrombosis, in which the clots can be found in the fetal circulation. These complications can occur due to multiple aetiologies specifically associated with the Virchow’s triad, involving stasis, endothelial injury, and hypercoagulability [7].

Pregnant women are one of the high-risk categories for COVID-19 infection given their circumstances during and after pregnancy compared to non-pregnant women. This is due to pregnant women experiencing immunologic and physiologic alteration, making them more susceptible to viral respiratory infections [8]. Without COVID-19, they are more likely to develop adverse pregnancy outcomes, e.g., preeclampsia and preterm labor. However, once infected with COVID-19, they are more likely to experience serious complications due to massive inflammation and endothelial damage, thereby triggering the coagulation cascade to be activated and thereby promoting thromboembolic complications.

As cases involving TE events in pregnant women have evolved, practitioners have utilized anticoagulation as the prophylaxis and initial treatment. Low molecular weight heparin (LMWH) is used as a thromboprophylaxis and treatment in pregnant women infected with COVID-19.

Since COVID-19 has created a serious threat to human health resulting in significant increases in morbidity and mortality worldwide, the scenario becomes considerably more serious when the virus infects pregnant mothers since they are already in a hypercoagulable state causing substantial inflammation and coagulation leading to the thromboembolic complications. These TE complications have been reported in many pregnant women with the outcomes being either survival or death. Therefore, the purpose of this study is to review the prevalence of TE events among pregnant women who are infected with COVID-19 as well as their preventive strategies.

## 2. Materials and Methods

A comprehensive structured literature search was performed based on the scientific online databases PubMed and ScienceDirect published from February 2020 to April 2022. The search included research papers, case series, and case reports with the following keywords: COVID-19, thromboembolic complications, pregnancy, prevalence, venous thromboembolism, pulmonary embolism, deep venous thrombosis, disseminated intravascular coagulation, and intervillous thrombosis. A search filter was used to only include articles published in the English language. The articles were screened by assessing the title and abstract for the inclusion and exclusion criteria. The inclusion criteria included research publications, original articles, case reports, and case series. General review, irrelevant research regarding thrombocytopenia, placenta as the subjects without details of the mothers, physician as the subjects instead of pregnant women, and fetal vessels were all excluded. The gathered articles were assessed for eligibility in which only studies with reported prevalence of TE events were included. The final gathered studies (*n* = 19) were then extracted into Microsoft Excel software for the data analysis.

The prevalence of TE complications among positive COVID-19 pregnant women was calculated by dividing the number of women who experience thromboembolism by the entire population of pregnant women involved in collected studies. Whereas the prevalence of TE complications reported from studies focusing on placenta were calculated by dividing the total number of those who develop placental thromboembolism by the total number of placentas from the collected studies.

## 3. Results

**Figure 1:**
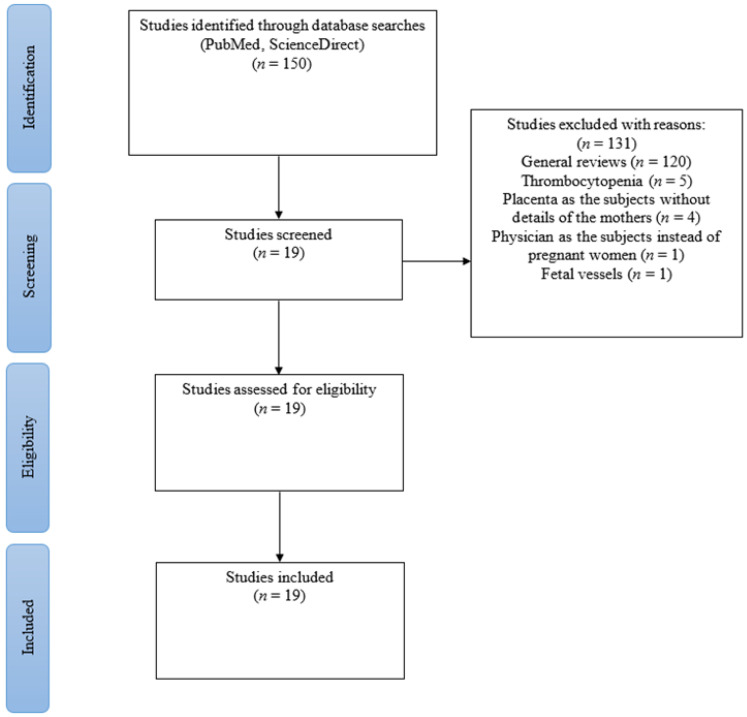
PRISMA chart.

The search identified 150 articles, which after the screening of titles, abstracts, and assessing for eligibility, only 19 studies were included (Figure 1). From the 19 studies, most [8,9,10,11,12,13,14,15,16,17,18,19,20,21,22,23,24,25,26] of them were (*n* = 5) case series [16,17,18,19,20] and case reports (*n* = 6) [21,22,23,24,25,26]. Seven studies [8,10,11,12,13,14,15] were performed retrospectively and one [9] was a systematic review. The studies were mostly conducted from 2018 until May 2021. These 19 studies involving 5249 participants included 5128 pregnancies and 121 placentas being examined for TE complications caused by SARS-CoV-2 virus.

According to Table 1 and Table 2, from the 19 studies included, 7 studies were conducted in North America [10,12,13,14,15,18,20], 6 in Europe [11,17,19,23,24,26], 1 in the Middle East [21], and others were each conducted in South Asia [22], East Asia [8], and South America [25], and one was a multicentre study [16]. Most studies were performed in adults (mean/median age ranging from 17 to 47 years). The majority (*n* = 4012) of the patients included were Hispanic (44.9%) and Non-Hispanic White (31.1%) with the mean/median age ranging from 28.9 to 30 years, while the remaining were Non-Hispanic Black (14.5%), Asian (1.37%), African-American (0.72%), and others (7.45%) [10,12,14,15,25]. 

Based on the 19 studies included, pregnant mothers have many comorbidities, e.g., diabetes mellitus type 1 and type 2 [13,15,23,24], gestational diabetes [15], hypothyroidism [13,17], hypertension [13,15], preeclampsia [13,23], and asthma [13,15,24] (Table 1 and Table 2). One case report [21] recorded in Iran presented a patient with clinical manifestations of loss of consciousness, double mydriasis, and tonic-clonic seizures (Table 1).

Laboratory findings (Table 3, Table 4 and Table 5) that were associated with TE events include D-dimer, fibrinogen and C-reactive protein (CRP) levels, as well as radiological imaging. Eight studies [9,11,16,17,22,23,25,26] showed elevated D-dimer level (1.8 to 34.47 mg/L) (Normal range (NR): 0.1–1.7 mg/L), five studies [8,11,16,23,26] for elevated CRP level ranging from 14 to 60 mg/L (NR: 1–10 mg/L), and four studies [16,21,25,26] for elevated fibrinogen ranging from 5.43 to 3530 g/L (NR: 1.5–4.2 g/L). In addition, seven studies [12,18,21,22,24,25,26] included radiological imaging and histopathology examination to confirm the diagnosis of TE complications. For instance, routine histopathology and complement staining for placental thrombosis, echocardiography, computerized tomography pulmonary angiogram (CTPA) and chest CT scan for PE, as well as fetal-placental magnetic resonance imaging, anatomopathological examination of placenta, and placenta pathology for IVT.

The most common TE complications within pregnant women (*n* = 5128) were DIC (*n* = 44, 0.86%), TE (*n* = 14, 0.27%), IVT (*n* = 9, 0.18%), PE (*n* = 6, 0.12%), CAC (*n* = 5, 0.10%), and DVT (*n* = 2, 0.04%). (Table 3, Table 4 and Table 5). Meanwhile, the prevalence of TE complications reported from studies focusing on placenta (*n* = 121) were IVT (*n* = 27, 22.3%), SCT (*n* = 9, 7.44%), and placental thrombosis (*n* = 5, 4.13%) (Table 3 and Table 4). TE complications that occur during pregnancy were DIC (*n* = 37, 33.3%), TE (*n* = 13, 11.7%), CAC (*n* = 3, 2.7%), and PE (*n* = 2, 1.8%), whereas cases that occur during the postpartum period were IVT (*n* = 36, 32.4%), SCT (*n* = 9, 8.1%), placenta thrombosis (*n* = 5, 4.5%), DVT (*n* = 2, 1.8%), PE (*n* = 2, 1.8%), and CAC (*n* = 2, 1.8%) (Table 1 and Table 2). From the 19 studies included, seven mothers [9,15,21,24] and two neonates died [21,25], whereas the others were discharged well [8,15,16,17,18,22,23,26] (Table 3, Table 4 and Table 5).

Only nine studies [9,11,14,16,17,21,22,24,26] reported thromboprophylaxis strategies among COVID-19 infected pregnant women with TE complications (Table 6). Most studies (*n* = 9) used anticoagulation, specifically LMWH such as Enoxaparin at prophylactic dose (40–60 mg once daily (OD)). Only one study [9] used therapeutic dose of anticoagulant for the prevention of TE complications.

## 4. Discussion

In this study, we investigate the global prevalence of COVID-19 positive pregnant women who experience TE complications. Findings showed that the prevalence of TE complications among pregnant women infected with COVID-19 is low with the most common form being DIC. LMWH (in variable dose) is frequently used to prevent TE complications in pregnant women.

According to the results obtained, the types of TE complications which developed among pregnant women with COVID-19 were DIC (*n* = 44, 0.86%), TE (*n* = 14, 0.27%), IVT (*n* = 9, 0.18%), PE (*n* = 6, 0.12%), CAC (*n* = 5, 0.10%), and DVT (*n* = 2, 0.04%), whereas complications that developed in the placenta were IVT (*n* = 27, 22.3%), SCT (*n* = 9, 7.44%), and placental thrombosis (*n* = 5, 4.13%). Most of the pregnant women were in the age range of 17 to 47 years old, with underlying comorbidities such as hypertension and diabetes mellitus and presented with TE complications more commonly in the postpartum rather than prepartum period.

Several explanations can be given to the observed findings. Both pregnancy and COVID-19 infection can increase the risk of TE complications. Pregnant women are already known to be in a hypercoagulable state physiologically, so they are more susceptible to COVID-19 due to their physiological alterations. This indirectly can increase the risk of dying due to COVID-19 [9,27]. Pregnant women experience a significant immunocompromised condition generated by the changes in the body’s cell-mediated immune response and inflammatory mechanisms [28]. Pro-inflammatory factors such as von Willebrand factor and fibrinogen are intended to prevent excessive blood loss after birth, but in certain women, this effect is exaggerated causing them to have an elevated risk of thrombosis [1]. Therefore, this hypercoagulable state increased the risk of TE events and morbidity by 4- to 6-fold, with higher risk in the post-partum period [28].

The SARS-CoV-2 virus causes endothelial injury and triggers the innate immune responses by activating the monocytes which are responsible for massive release of proinflammatory cytokines and also platelets activation [29]. Then, the ACE2 receptor on the endothelium surface binds to SARS-CoV-2 virus resulting in conversion of Angiotensin I (ATI) to Angiotensin II (ATII). This ATII pathway causes inflammation by increasing the levels of pro-inflammatory cytokines and chemokines such as Interleukin-6 (IL-6), IL-8, and tumor necrosis factor-α (TNF-α). The cytokines enters the circulation and attract T-lymphocytes and monocytes, causing inflammatory injury to the virally infected cells [30]. Thus, overproduction of ATII will cause the overproduction of cytokines [31].

The pathophysiology of thromboembolism can be aggravated by inflammation [32]. This is because inflammation, particularly at the blood vessel walls, can trigger the activation of the coagulation pathway by inducing the tissue factor (TF). When blood vessels become inflamed, endothelial cells, platelets, and cytokines are generated, triggering the production of TF. By increasing the expression of TF, these mediators can increase the procoagulant state. TNF-α can activate Factor II, causing activation of the extrinsic coagulation cascade [33].

Apart from that, inflammation can increase hypercoagulability by stimulating TF pathways and inducing NETosis [33]. In response to infection, it creates neutrophils extracellular traps (NET), which allows them to trap and destroy the invading virus. The production of NET, which induces fibrin production and deposition in the blood vessels, causes a reaction known as NETosis [32]. As a result, the increased amount of NET promotes synthesis of fibrin, which increases the risk of thrombus and thereby amplifies the hypercoagulable condition state [33].

Overall, SARS-CoV-2 increases NET’s formation, cytokine overproduction, and hyperinflammation, in which all of these will consequently cause coagulopathy [31]. So, once the virus infects pregnant women, it will cause a massive inflammatory reaction since they are already in a hypercoagulable state, and this will further increase the risks of TE complications.

Our findings suggest that DIC is the most common type of TE complication among COVID-19 positive pregnant women (*n* = 44, 0.86%). This can be due to the fact that DIC is secondary to many clinical conditions such as sepsis and septic shock as well as obstetric conditions such as eclampsia [17]. The term sepsis refers to the body’s severe reaction towards a life-threatening infection, e.g., bacterial infection or pneumonia, and can lead to death [34]. To fight the infection, the immune system goes into overdrive, releasing chemicals into the bloodstream. This sets off a chain reaction resulting in serious inflammatory reaction throughout the body [35]. The SARS-CoV-2 virus can induce pneumonia in individuals which can lead to viral sepsis. As a result, it can trigger concurrent activation of coagulation and fibrinolytic cascade, which leads to coagulopathy [16].

Other than DIC, other complications occurring in pregnancies were TE (*n* = 14, 0.27%), PE (*n* = 6, 0.12%), CAC (*n* = 5, 0.10%), and DVT (*n* = 2, 0.04%). This is plausible given that pregnant women are at a higher risk of both venous and arterial thrombosis during pregnancy, but the risk of VTE is greater than the risk of arterial thrombosis, which is 4- to 5- folds compared to arterial (3- to 4-fold) [5,36]. From our findings, the risk of TE complications is higher (*n* = 56) in the postpartum period compared to the prepartum period (*n* = 55) and studies have shown approximately 2 out of every 1000 births of pregnant women will experience TE events [5]. Since COVID-19 infection is highly prothrombotic and linked to a unique type of coagulopathy, it can cause systemic inflammation and CAC. CAC develops because of overstimulation of the inflammatory cascade, leading to endothelial and platelet activation [37]. Additionally, the presence of a hypercoagulation state triggered by the deep and complex inflammatory response to the virus as well as hypercoagulability state of pregnant women alters the coagulation cascade in the body in a condition also known as ‘thrombo-inflammation’ [38].

Furthermore, there were also TE complications in placenta, which were IVT, SCT, and placental thrombosis. IVT is a specific form of thrombosis that occurs in the placenta and is characterised by the presence of blood clots in the intervillous space, and it is found next to the chorionic plate (subchorionic), in the centre of the parenchymal mass, or adjacent to the basal plate [7]. SCT, on the other hand, occurs due to the deposition of fibrin plaque in the subchorionic space in the placenta, and lastly, placental thrombosis can occur in fetal circulation. These pathological lesions are linked to the Virchow’s triad (venous or circulatory stasis, hypercoagulability, and vein damage). Stasis of maternal blood decreases blood flow in the placenta and enables the accumulation of procoagulant proteases, e.g., thrombin and fibrinogen, which may overcome the local anticoagulant pathways and thereby induce thrombosis [39]. It might also be related to irregular blood flow, which can occur when IVT, SCT, and placental thrombosis develop as a result of direct compression during labour or acute villous oedema, where substantial deformation of the villi creates local stasis and thrombus [7]. Other than that, it might be attributed to maternal hypercoagulability, in which thrombi form spontaneously, most frequently in low-flow regions.

There are several other studies investigating the TE complications among pregnant women infected with COVID-19. According to a case control study by Resta et al. [40], they discovered thrombi in fetal vessels in 16 out of 71 cases of pregnant mothers infected with COVID-19. Moreover, Ko et al. [41] found an adjusted risk ratio of 2.7 for TE complications from 6550 COVID-19 positive pregnancies. Another study by Levitan et al. [42] reported that 15 out of 65 pregnant women infected with COVID-19 developed intervillous thrombosis, whereas 18 of the 74 placentas from pregnant women who tested positive for COVID-19 in a study by Zhang et al. [43] developed thrombosis.

There are a variety of risk factors that might increase the chances of getting thromboembolism. Damage to a vein caused by a fracture, a significant major injury, or major surgery involving the pelvis, hip, or legs can all raise the risk of TE complications. Other factors that might increase the risk of TE include pregnancy, a family history of VTE, birth control pills, and increasing age [44].

Thromboembolism is not age specific. According to the 19 studies included [8,9,10,11,12,13,14,15,16,17,18,19,20,21,22,23,24,25,26], a majority of the studies were performed in adults with mean/median age ranging from 17 to 47 years. However, TE complications frequently occurred among pregnant women in the age range of 29 (five studies) [8,14,15,22,24].

It is widely known that D-dimer levels rise steadily during pregnancy and increased D-dimer concentrations indicate the activation of the coagulation cascade and reflect poor prognosis [11,45]. It was shown that pregnant COVID-19 patients had higher D-dimer levels than pregnant women who were not infected, which suggest increased risk of TE complications [45]. This can also be supported by a study by Wang et al. [45], which found that D-dimer levels in 27 pregnant women with COVID-19 infection ranged from below 3 µg/mL to over 5 µg/mL, all of which were beyond the normal range and indicated a higher risk of TE consequences. From the results obtained, 8 out of 19 studies showed elevated D-dimer levels, with values ranging from >1.8 to 34.37 mg/L. Moreover, CRP, a pentameric protein level, also rises in response to inflammation, and thus enhances the thrombotic response to vascular damage. Elevated CRP appears to be a key mechanistic relationship between inflammation and thrombosis since inflammation upregulates CRP expression. It has also been shown that elevated plasma fibrinogen reflects an inflammatory state and increases the risk of VTE because high fibrinogen indicates enhanced blood clot formation [28,46]. The findings showed that both CRP and fibrinogen values were increased, with values ranging from 14 to 60 mg/L and 5.43 to 3530 g/L, respectively.

From the 19 studies included [8,9,10,11,12,13,14,15,16,17,18,19,20,21,22,23,24,25,26], only 9 [9,11,14,16,17,21,22,24,26] reported the thromboprophylaxis used to prevent TE complications. Most of them used LMWH such as Enoxaparin at prophylactic dose (40 mg OD) [47]. According to The International Society of Thrombosis and Haemostasis (ISTH) and the Scientific and Standardization Committee (SSC) of the ISTH [48,49], a prophylaxis dose of LMWH for VTE is recommended to prevent TE complications in the absence of contraindications. In general, heparin-based drugs are typically the preferred anticoagulants during pregnancy since they do not cross the placenta or penetrate breast milk, hence it is safe to use during pregnancy. Apart from that, they also do not induce any anticoagulant effect in the newborns [3,50,51], hence showing favourable safety profile to this population.

## 5. Conclusions

The prevalence of TE complications among pregnant women infected with COVID-19 is low (<3%) with DIC and DVT as the most and least common type of TE complications, respectively. LMWH is frequently used as a thromboprophylaxis agent. Further research is required to detect the prevalence of TE complications and optimal management of thromboembolic complications in the future.

## Figures and Tables

**Table 1:** Demographic characteristics of case reports/case series reporting thromboembolic (TE) complications among pregnant women infected with COVID-19.

(a) **Author’s Name** (b) **Country**	Study Design	(a) **Number of Patients Included (*n*)** (b) **Maternal Age (Years Old) (Mean/Median/Range)** (c) **Ethnicity (*n*)**	Term of Pregnancy during Admission (Prepartum/Postpartum/Gestational Age)	Term of Pregnancy during Thrombotic Event (Prepartum/Postpartum)	Comorbidities/ Medical History	Clinical Presentations
(a) Koumoutsea, E. V., et al. [16] (b) Canada & France	Case series	(a) 2 mothers (b) 23 and 40 (c) -	Prepartum	Prepartum	-	-
(a) Skalska-Swistek, M., et al. [17] (b) Poland	Case series	(a) 2 mothers (b) 25 and 36 (c) -	34–35 weeks	Prepartum	Hypothyroidism diagnosed in first trimester	-
(a) Mulvey, J. J., et al. [18] (b) United States of America	Case series	(a) 5 placentas (b) 26–40 (range) (c) -	Prepartum	Postpartum	-	-
(a) Menter, T., et al. [19] (b) Switzerland	Case series	(a) 5 mothers (b) 27–39 (range) (c) -	Prepartum	Postpartum	-	-
(a) Shanes, E., et al. [20] (b) Chicago	Case series	(a) 15 placentas (b) 23–41 (range) (c) -	Postpartum	Postpartum	-	-
(a) Goudarzi, S., et al. [21] (b) Iran	Case report	(a) 1 mother (b) 22 (c) -	30 weeks 5 days	Prepartum	-	Loss of consciousness, double mydriasis, tonic-clonic seizure
(a) Kripalani, Y., et al. [22] (b) India	Case report	(a) 1 mother (b) 29 (c) -	38 weeks 1 day	Postpartum	-	-
(a) Mongula, J. E., et al. [23] (b) Netherlands	Case report	(a) 1 mother (b) 27 (c) -	31–32 weeks	Prepartum	Type 1 diabetes with low dose insulin, pre-eclampsia in previous pregnancy	-
(a) Ahmed, I., et al. [24] (b) United Kingdom	Case report	(a) 1 mother (b) 29 (c) -	29 weeks	Postpartum	Type 2 diabetes mellitus, renal tubular acidosis, asthma, vitamin D deficiency	-
(a) Marinho, P. S., et al. [25] (b) Brazil	Case report	(a) 1 mother (b) 33 (c) Black	34 weeks 4 days	Postpartum	A history of previous bariatric surgery (Roux-en-Y gastric bypass)	-
(a) Martinelli, I., et al. [26] (b) Milan	Case report	(a) 1 mother (b) 17 (c) -	29 weeks	Prepartum	-	-

**Table 2:** Demographics characteristics of retrospective/prospective/cohort studies reporting thromboembolic (TE) complications among pregnant women infected with COVID-19.

(a) **Author’s Name** (b) **Country**	(a) **Study Design** (b) **Duration of Data Collection**	(a) **Number of Patients Included (*n*)** (b) **Maternal Age (Years Old) (Mean/Median/Range)** (c) **Ethnicity (*n*)**	**Term of Pregnancy during** **Admission** **(Prepartum/Postpartum/** **Gestational Age)**	**Term of Pregnancy** **during Thrombotic Events** **(Prepartum/Postpartum)**	**Comorbidities/Medical History of the Mothers**
(a) Gulersen, M., et al. [10] (b) New York	(a) Retrospective cohort study (b) 9 April–27 April 2020	(a) 50 placentas (b) 30 (median) (c) Non-Hispanic Black (12) Non-Hispanic White (14) Asian (7) Hispanic (9) Others (8)	Third trimester	Postpartum	-
(a) Wu, Y. T., et al. [8] (b) China	(a) Retrospective study (b) 30 January–10 March 2020	(a) 29 mothers (b) 29.59 (mean) (c) -	35–41 weeks	Postpartum	-
(a) Pereira, A., et al. [11] (b) Spain	(a) Retrospective study (b) 14 March–14 April 2020	(a) 60 mothers (b) 22–43 (range) (c) -	Prepartum	Postpartum	-
(a) Jani, S., et al. [12] (b) Detroit	(a) Retrospective study (b) 11 March–31 July 2020	(a) 34 mothers (b) 26.3 (mean) (c) African Americans (29) Asian (3) Middle Eastern (1) Caucasian (1)	Prepartum	Postpartum	-
(a) Smithgall, M. C., et al. [13] (b) New York	(a) Retrospective study (b) 23 March–29 April 2020	(a) 51 placentas (b) 19–47 (range) (c) -	Postpartum	Postpartum	Obesity, Hypertension, Pre-eclampsia, Diabetes, Hypothyroidism, Asthma
(a) Metz, T. D., et al. [14] (b) America	(a) Observational cohort study (b) 1 March–31 July 2020	(a) 1219 mothers (b) 29 (mean) (c) Non-Hispanic Black (275) Non-Hispanic White (181) Hispanic (651) Others (112)	Prepartum	Prepartum	-
(a) Litman, E. A., et al. [15] (b) United States	(a) Observational cohort study (b) 1 January 2019–31 May 2021	(a) 2708 mothers (b) 28.9 (mean) (c) Non-Hispanic White (1052) Non-Hispanic Black (293) Hispanic (1142) Asian (45) Others (176)	Prepartum	Prepartum	Asthma, Autoimmune disease, Chronic kidney disease, Diabetes Mellitus, Gestational diabetes mellitus, Gestational hypertension, Hypertension, Major mental illness, Pulmonary disease

**Table 3:** Laboratory findings, Prevalence, and Outcomes of case reports/case series reporting thromboembolic (TE) complications among pregnant women infected with COVID-19.

(a) **Author’s Name** (b) **Country**	Laboratory Findings				Prevalence of TE Complications (*n*)					Outcomes (Death/Alive) (*n*)
	D-Dimer (mg/L) NR: 0.1–1.7	CRP (mg/L) NR: 0.1–10	Fibrinogen (g/L) NR: 1.5–4.2	Radiological Imaging Confirmation	DIC	PE	IVT	CAC	Placental Thrombosis	
(a) Koumoutsea, E. V., et al. [16] (b) Canada & France	>20	>37	Elevated	-	-	-	-	2	-	All recovered
(a) Skalska-Swistek, M., et al. [17] (b) Poland	>34.47	-	3.1	-	2	-	-	-	-	All discharged
(a) Mulvey, J. J., et al. [18] (b) United States of America	-	-	-	Routine histopathology & complement staining: Frank thrombosis of fetal chorionic plate vessels	-	-	-	-	5	Mothers were discharged, unknown outcome for fetals
(a) Menter, T., et al. [19] (b) Switzerland	-	-	-	-	-	-	1	-	-	-
(a) Shanes, E. D., et al. [20] (b) Chicago	-	-	-	-	-	-	6	-	-	No maternal and neonatal death
(a) Goudarzi, S., et al. [21] (b) Iran	High	-	810	-	-	1	-	-	-	Maternal death and fetal death in mother’s uterus
(a) Kripalani, Y., et al. [22] (b) India	Elevated (1.8)	-	-	CTPA: signs of a hypodense filling defect, suggestive of pulmonary thromboembolism in theanterior basal and lateral basal segmental and subsegmental branches of the right lower lobar pulmonary artery	-	1	-	-	-	Discharged
(a) Mongula, J. E., et al. [23] (b) Netherlands	>9.4	>14	0.7–4.2	-	-	-	-	1	-	Recovered and discharged
(a) Ahmed, I., et al. [24] (b) United Kingdom	-	-	-	CTPA: revealed right lower lobar pulmonary embolism.	-	1	-	-	-	Death
(a) Marinho, P. S., et al. [25] (b) Brazil	17.22	-	3530	Fetal-placenta Magnetic Resonance Imaging: The placenta had a posterior uterine wall insertion, large and dilated vessels with massive thrombosis	-	-	1	-	-	Death
(a) Martinelli, I., et al. [26] (b) Milan	>15.8	>28.1	>5.43	Chest CT scan:segmental pulmonary embolus in the right superior lobe,	-	1	-	-	-	Discharged

Abbreviation: NR, normal range; CRP, C-reactive protein; DIC, disseminated intravascular coagulation; PE, pulmonary embolism; IVT, intervillous thrombosis; CAC, COVID-19 associated coagulopathy.

**Table 4:** Laboratory findings, Prevalence, and Outcomes of retrospective/prospective/cohort studies reporting thromboembolic (TE) complications among pregnant women infected with COVID-19.

(a) **Author’s Name** (b) **Country**	Laboratory Findings			Cases of TE Complications (*n*)							Outcomes (Death/Alive) (*n*)
	D-Dimer (mg/L) NR: 0.1–1.7	CRP (mg/L) NR: 0.1–10	Radiological Imaging Confirmation	DIC	PE	DVT	TE	IVT	SCT	CAC	
(a) Gulersen, M., et al. [10] (b) New York	-	-	-	-	-	-	-	13	-	-	-
(a) Wu, Y. T., et al. [8] (b) China	-	22.2	-	-	-	-	-	-	-	2	All discharged
(a) Pereira, A., et al. [11] (b) Spain	>1.9	>60	-	-	-	2	-	-	-	-	No maternal death
(a) Jani, S., et al. [12] (b) Detroit	-	-	Placenta pathology: Small isolated intervillous thrombi were seen in seven (21%) placentas	-	-	-	-	7	-	-	-
(a) Smithgall, M. C., et al. [13] (b) New York	-	-	-	-	-	-	-	8	9	-	-
(a) Metz, T. D., et al. [14] (b) America	-	-	-	-	-	-	8	-	-	-	-
(a) Litman, E. A., et al. [15] (b) United States	-	-	-	35	-	-	5	-	-	-	Discharged home (2610) Post-acute care (37) Death (3) Rehab (8) Hospice (1)

Abbreviation: NR, normal range; CRP, C-reactive protein; DIC, disseminated intravascular coagulation; PE, pulmonary embolism; DVT, deep venous thrombosis; TE, thromboembolism; IVT, intervillous thrombosis; SCT, subchorionic thrombus; CAC, COVID-19 associated coagulopathy.

**Table 5:** Systematic review reporting thromboembolic (TE) complications among pregnant women infected with COVID-19.

Author’s Name	Study Design	Number of Patients Included (*n*)	Laboratory Findings		Cases of TE Complications (*n*)			Outcome (Death/Alive) (*n*)
			D-Dimer (mg/L) NR: 0.1–1.7	Fibrinogen (g/L) NR: 1.5–4.2	DIC	PE	TE	
Servante, J., et al. [9]	Systematic review	1063 mothers	>19.06	<2.2	7	2	1 (Inferior vena cava)	2 DIC cases reported dead

Abbreviation: NR, normal range; CRP, C-reactive protein; DIC, disseminated intravascular coagulation; PE, pulmonary embolism; TE, thromboembolism.

**Table 6:** Thromboprophylaxis used among COVID-19 infected pregnant women with TE complications.

Author’s Name	Cases of TE Complications (*n*)					Types & Dose of Thromboprophylaxis
	DIC	PE	DVT	TE	CAC	
Servante, J., et al. [9]	7	2	-	1 (Inferior vena cava)	-	Enoxaparin 40 mg OD (*n* = 3) Therapeutic anticoagulation (*n* = 1)
Pereira, A., et al. [11]	-	-	2	-	-	LMWH (no information on type and dose)
Metz, T. D., et al. [14]	-	-	-	8	-	Prophylactic anticoagulant in 5 out of 8 TE patients (no information on type and dose)
Koumoutsea, E. V., et al. [16]	-	-	-	-	2	LMWH prophylactic dose
Skalska-Swistek, M., et al. [17]	2	-	-	-	-	LMWH prophylactic dose 2nd patient
Goudarzi, S., et al. [21]	-	1	-	-	-	No information
Kripalani, Y., et al. [22]	-	1	-	-	-	Enoxaparin 60 mg OD
Ahmed, I., et al. [24]	-	1	-	-	-	Enoxaparin (no information on dose)
Martinelli, I., et al. [26]	-	1	-	-	-	Enoxaparin 40 mg OD

Abbreviation: DIC, disseminated intravascular coagulation; PE, pulmonary embolism; DVT, deep venous thrombosis; TE, thromboembolism; CAC, COVID-19 associated coagulopathy; VTE, venous thromboembolism; LMWH, low molecular weight heparin.

## Data Availability

All data have been included in this manuscript.
